# Mental Imagery Skills in Competitive Young Athletes and Non-athletes

**DOI:** 10.3389/fpsyg.2020.00633

**Published:** 2020-04-17

**Authors:** Donatella Di Corrado, Maria Guarnera, Claudia Savia Guerrera, Nelson Mauro Maldonato, Santo Di Nuovo, Sabrina Castellano, Marinella Coco

**Affiliations:** ^1^Department of Human and Social Sciences, Kore University, Enna, Italy; ^2^Department of Formative Processes, University of Catania, Catania, Italy; ^3^Department of Neuroscience and Reproductive and Odontostomatological Sciences, University of Naples Federico II, Naples, Italy; ^4^Department of Biomedical and Biotechnological Sciences, University of Catania, Catania, Italy

**Keywords:** mental imagery, children, athletes, sport, cognitive performance

## Abstract

Mental imagery is a fully immersive multi-sensory procedure that associates as numerous senses to create a mental image and process it without the presence of external stimuli. In the sport situation, imagery has been designated as the state in which people imagine themselves while effecting abilities to deal with the forthcoming duty or improve performance. Methodical analyses have revealed that imagery increases performance in motor tasks. This study aims at observing not the vividness of image but the cognitive abilities useful for the inspection, maintenance, generation, and manipulation of dissimilar classes of images, investigating modifications in mental imagery skills in competitive athletes and non-athletes. Participants were competitive athletes (*n* = 48) and non-athletes (*n* = 48) between the ages of 8 and 13 years (*M*_age_ = 10.50, *SD* = 1.73). The athletes had a minimum of 5 years of training skill in the sport. They completed the Mental Imagery Test (MIT). Competitive athletes showed higher scores on mental imagery skills than non-athletes. The study contributed to increase the exploration in the definite area of mental imagery, supplying an added support for the pluridimensional nature of mental imagery and for its practicality in motor and sport sciences.

## Introduction

Mental imagery is the reproduction of perceptual experience (Kosslyn et al., 2001; Pearson, 2007) across multisensory ways and the processing of images in the absence of external stimuli. Mental imagery is a significant element in human functioning.

In the sport situation, imagery has been designated as the state in which people imagine themselves while effecting abilities to deal with the forthcoming duty or improve performance. Imagery may be a consequence from both thoughtful and unconscious recall procedures; an individual sees an image, or experiences a movement as an image, without experiencing the real thing through a process. Imagery plays a significant role in this context, improving performance in motor tasks (Di Corrado et al., 2014, 2019).

It is usually assessed in relations of its mental and emotional characteristics, as well as motivational competence (Cumming and Williams, 2012). Owing to the gains of imagery, it is, nowadays, included in numerous mental skills line-ups, in addition to physical preparation.

Various overlying terms have been utilized to refer to several sides of mental imagery in sports (Hale et al., 2005; Cumming and Ramsey, 2009; Collet et al., 2013). Morris et al. (2005) proposed that a further complete description of sports imagery is “*the intentional or unintentional conception or regeneration of an experience produced from memorial information, concerning quasi-affective and quasi-sensorial features that may occur in the absence of the tangible stimulus antecedents usually associated with the real experience*” (p. 19).

Within the mental mode, kinesthetic (i.e., perceptual involvement of the body while executing a movement) and visual (i.e., what a person sees) are the most usual sensory tested mentally approaches of creating images (Guarnera et al., 2016).

However, imagery is a complex construct. In the several explorations of mental imagery, researchers have investigated equally *imagery use* and *imagery ability*. The capability of creating vivid images is different from the competence in controlling and manipulating the created images (Morris et al., 2005). Cognitive psychology designed instruments to measure imagery ability into two aspects: controllability, i.e., “*manipulate mentally an image in precise way*” (Guillot and Colle, 2010; Pirrone and Di Nuovo, 2014), and vividness, i.e., “*the sensory richness of an image*” (Murphy, 2005). Murphy and Martin (2002) defined imagery use as the “*use of imagery to reach a variety of cognitive, behavioral, and affective changes*” (p. 418). Hall et al. (1998) underline that images address distinct motivational and cognitive aspects, both on a common and definite level. Consequently, differentiations in the use of imagery occur: cognitive specific (e.g., movements), cognitive general (e.g., tactics), motivational general, motivational specific (e.g., aims), motivational general–affective (e.g., anxiety), and motivational general–mastery (e.g. self-confidence).

In addition to the aforementioned considerations, the model of imagery suggested by Kosslyn et al. (2006) established which cognitive procedures are required in order to generate the configurations of behavior related with the everyday use and experience of mental imagery (Slotnick et al., 2012). These processes consist of generation, inspection, maintenance, and manipulation of visual images, in the lack of visual input. In detail, generation is the ability to generate mental representations deprived of a perceived stimulus. Maintenance is the capacity to maintain images in the short-term memory. Inspection describes the capacity to explore a generated image to interpreting a spatial property or an object-based characteristic of the image. Manipulation is the capacity to modify a mental image in some manner. Individuals can manipulate, transform, or rotate objects in visual imagery much like real objects, in their mind (Pearson and Kosslyn, 2013). This component of “controllability” of the images has been less studied in athletes.

Although previous investigation on mental imagery has principally concentrated on adult athletes, fewer studies have examined the young athlete population. Ashby (1983) have showed that imagery ability does not grow completely until 7 years of age. Parker and Lovell (2009) discovered high improvement in imagery ability in athletes between 7 and 17 years of age. Regarding the imagery use, previous studies showed that young athletes, competing at different levels, obviously use all components of imagery (Munroe-Chandler et al., 2007a; Parker and Lovell, 2012).

Comparing athletes’ imagery with that of non-athletes, Isaac and Marks (1994) investigated whether distinctions in movement and visual imagery vividness can be assessed in 16 different sporting disciplines. Results showed that elite athletes had significantly higher vivid imagery than non-athlete controls. Williams and Davids (1995) compared the soccer knowledge base of three groups of participants: highly skilled, lower skilled, and controls (who had never played the game). Results showed that the highly skilled participants scored considerably higher than the others did on the ability to recognize and recall soccer movements. Moreau et al. (2011) investigated whether the level of expertise in a particular sport is related to better motor performance and spatial abilities, such as mental rotation, in particular. The findings indicated that young elite athletes reported consistent high performance in the spatial ability.

In a research on high school athletes, Godoy-Izquierdo et al. (2007) described more levels of imagery in elite athletes compared with non-elite ones. Mohammadzadeh and Sami (2014) investigated some psychological skills such as motivation, imagery, arousal, focus, self-confidence, and goal setting of elite and non-elite volleyball players (*N* = 60). Regarding imagery as the usage of all the senses to recreate or make an experience in mind, the results indicated that elite volleyball players had superior performance than non-elite ones.

Nevertheless, little investigation has been conducted on younger athletes comparing imagery—i.e., perception without present stimuli—with other cognitive abilities (e.g., spatial perception and visual memory).

The aim of the current study is to investigate the ability of children to engage in visual and spatial mental imagery (image maintenance, image inspection, image generation, and image manipulation), and to compare these processes between athletes and non-athletes (8–13 years of age). This age range has been selected as it is recommended that children aged 7 and over can form kinesthetic and transformational images (Piaget and Inhelder, 1971). In fact, although in disagreement with Piaget’s theory, much research has demonstrated that under certain conditions, children younger than 7 years of age can transform visual mental images (Quaiser-Pohl et al., 2010; Guarnera et al., 2017, 2019). There is general agreement that children over 7 years of age improve their imagery ability, as they move from perceptual to conceptual activity (Munroe-Chandler et al., 2007b).

We expected athletes to show greater ability in controlling mental images than non-athletes.

## Materials and Methods

### Participants and Procedure

The participants Were competitive athletes (*n* = 48) and non-athletes (*n* = 48) Between the ages of 8 and 13 years. the groups Were matched so they Had the same mean and standard deviation (*M*_age_ = 10.50, *SD* = 1.73, *t* = 0).

The athletes had a minimum of 5 years of training skill in the sport, and they were all associates of sport clubs. These athletes had different competitive sport backgrounds (e.g., soccer, karate, gymnastics, dance, and handball). Their hours of exercise per week ranged from 3.0 to 21.5 h (*M* = 7.39, *SD* = 3.41). Forty-eight students who had no experience in any sport represented control participants. Participants did not obtain before mental abilities or imagery training. Measurements were carried in clusters of four participants. Athletes were tested in an isolated place near the training accommodations. The control group was from dissimilar classes and was tested in a separate place near the school at the end of the educational activities. Prior to the beginning of the study, ethical approval was granted from the first author’s university ethics committee. The study obtained ethical permission from the University Enna Kore Internal Review Board for psychological research (September 26, 2019).

Before starting the protocol, parents signed a consent form, and were informed about the processes of the study and the anonymity of their answers, in accordance with the Declaration of Helsinki.

### Measures

#### Imagery Assessment

A sequence of tasks evaluating mental imagery skills including maintenance, inspection, generation, and manipulation of different categories of images was administered. Eight tasks are incorporated in the Mental Imagery Test (MIT) and standardized for usage in childhood (Di Nuovo et al., 2014):

##### Visualizing letters

The individuals are invited, without seeing the stimuli and spending only imagination, to say which upper-case letters have curled parts (e.g., A, P, or R; not L, M, or N).

##### Brooks “F” test

With their imagination, the individuals are asked to walk along the delineation of a large upper-case letter F earlier viewed for 30 s on a reproduced card. The individual is asked to say whether the limits encountered when moving from the lower left corner in a counter-clockwise direction are external or internal.

##### Clock

The task necessitates imagining a clock with hands indicating 10 min past 10:00, then imagining the clock reproduced in a mirror and saying what time the mirrored clock will show after 10 min.

##### Cube

The image of a large cube is shown for 30 s; it is collected of nine small cubes per face (3 × 3), and the external faces are colored. After the stimulus is detached, the subject is asked to state how many small cubes have three, two, one, or none external (colored) faces.

##### Subtraction of parts

A digital display with the number 88 collected of small segments is shown for 10 s. Then, another digital display with designated segments of a two-digit number is shown for 10 s. The individual is asked to imagine what two-digit number will remain after deducting the parts of the new figure from the figure with all digits seen beforehand.

##### Mental exploration of a map

The individual is presented with a map of an island, with a house, a church, a lake, and a wood located on it. The instructions ask to look with attention at the map, staring at the distances among the elements located. After the map is detached, the individual is asked to answer four questions about the comparative distance between couples of the earlier seen components.

##### Imagined paths

The individual is asked to imagine a small ball moving in dissimilar directions, succeeding a suggested path in the imagined space, and saying if at the end of the route the ball will end up above or below the initial point, or at the similar level.

##### Mental representation of shapes of objects

The individual hears the names of 20 concrete objects (e.g., bottle, pizza, candle, tower, and bed) and is asked to imagine them and select if the object has a taller or larger form.

A total score of mental imagery can be found by the sum of the scores in the single subtests. Cronbach’s alpha for this score of imagery is 0.75. A confirmatory factor analysis showed the monodimensionality of the measure containing the eight tasks, in a sample of 556 participants aged 8 to 13 years (chi-square = 186.28, *p* < 0.01; Root Mean Square Error of Approximation (RMSEA) = 0.09, *p* < 0.01; Comparative Fit Index (CFI) = 0.90; Standardized Root Mean (SRM) = 0.05; Goodness of Fit Index (GFI) = 0.93 (Di Nuovo et al., 2014).

The selection of the tasks, resulting from the preceding literature on mental imagery, was designed at expressing all the functions implicated in imagery, agreeing to the classification proposed by Pearson et al. (2013).

#### Comparison Tasks

With the aim of discriminating mental imagery abilities from those based on memory and visual perception—without necessitating active generation and/or alteration of mental images—some other cognitive standardized tasks are proposed.

##### Forward and backward digit span

The individual hears gradually increasing digit series, and has to repeat them in direct or backward order. Verbal working memory is implicated in these attentive tasks.

##### Memory of objects and of position of object*s*

The individual, after seeing seven concrete objects for 30”, is invited to evoke as many of them as possible in a free recall. Subsequently, the individual has to recall the position in a matrix of six objects before seen for 30”.

##### Visual–spatial memory test

Individuals are presented for 10” with a matrix in which a number of cells are completed and, after, are required to recall where they were located using a blank matrix. Six matrices are presented, and completed cells increase gradually from 2 to 4.

The tasks of memory of position, for both objects and matrices, necessitate a passive maintenance of spatial position in working memory and, consequently are useful to differentiate this skill from active elaboration of mental images.

##### Mirror reproduction of spatial position

The individual is requested to copy a model in a mirror position, though really seeing the model. This task of copy includes spatial perception without active alteration of images.

### Statistical Analyses

Differences between the two groups of participants were assessed by *t*-test comparisons. Moreover, Pearson correlational analyses were completed to explore possible existing relationships between mental imagery abilities, and memory and visual perception within each group of participants. Statistical analyses were performed using SPSS version 23 Statistical Software Package for Windows (SPSS Inc., Chicago, IL, United States).

## Results

Preliminary, for the global score of imagery, we verified that the general difference due to the gender was non-significant: males’ mean = 53.27, *SD* = 13.74; females’ mean = 53.15, *SD* = 12.24; *t* = 0.05; *p* = 0.96. For this reason, the subsequent analyses were carried out without taking into account gender.

The analyses of differences in the performances of controls and athletes in the specific tasks are shown in Table 1.

**TABLE 1:** *T*-test results.

**Variables**		**Controls**	**Athletes**	***T***	**P**
**Mental imagery abilities**					
Visualizing letters	M	10.02	11.88	–6.22	<0.001
	SD	2.04	0.33		
Brooks “F” test	M	4.10	7.44	–6.43	<0.001
	SD	2.42	2.66		
Clock	M	1.29	0.08	5.00	<0.001
	SD	1.62	0.40		
Cube	M	1.15	1.71	–1.40	0.17
	SD	1.53	2.33		
Subtraction of parts	M	5.04	10.04	–8.65	<0.001
	SD	3.23	2.37		
Mental exploration of a map	M	4.00	5.23	–4.64	<0.001
	SD	1.49	1.08		
Imagined paths	M	4.29	7.48	–6.03	<0.001
	SD	2.56	2.62		
Mental representation of shapes of objects	M	14.13	18.54	–7.24	<0.001
	SD	4.12	0.94		
**Memory and visual perception**					
Forward digit-span	M	3.87	5.10	–4.07	<0.001
	SD	1.95	0.75		
Backward digit-span	M	1.88	3.79	–7.56	<0.001
	SD	1.45	0.99		
Visual–spatial memory test	M	2.29	15.35	–16.39	<0.001
	SD	2.93	4.68		
Memory of objects	M	5.12	6.63	–5.73	<0.001
	SD	1.67	0.70		
Memory of position of objects	M	6.15	9.56	–6.30	<0.001
	SD	3.05	2.20		
Mirror reproduction of spatial position	M	9.58	15.31	–6.38	<0.001
	SD	5.80	2.25		

Results showed high significant differences in all mental imagery abilities (except the “cube” test) and, in memory and visual perception with athletes. The only task showing a different trend is “clock,” the performance being better in the control group.

In addition, the difference in the total score of imagery is highly significant: for the athletes group, mean = 62.40, *SD* = 7.13; for the controls group, mean = 44.02, *SD* = 10.74; *t* = −9.87, *p* < 0.001.

Correlational analyses between imagery tasks and perceptual and memory tests were performed separately for the two groups of athletes and controls (Tables 2, 3). Bonferroni confidence interval adjustments were used throughout.

**TABLE 2:** Pearson correlation matrix between imagery subtest and perceptual and memory tasks—athletes.

**Athletes**	**Forward digit-span**	**Backward digit-span**	**Visual–spatial memory test**	**Memory of objects**	**Memory of position of objects**	**Mirror reproduction of spatial position**
Visualizing letters	0.31*	0.11	0.27	0.16	0.27	0.08
Brooks “F” test	0.19	0.13	0.38**	0.01	0.27	0.19
Clock	0.25	0.15	0.14	0.11	0.14	–0.08
Cube	–0.10	0.32*	–0.08	–0.07	–0.09	0.15
Subtraction of parts	0.32*	0.22	0.05	0.32	0.14	0.37**
Mental exploration of a map	0.05	0.07	–0.02	0.34*	0.16	–0.10
Imagined paths	0.37**	0.34*	0.22	0.22	0.00	0.42***
Mental representation of shapes of objects	0.25	0.37**	0.21	0.22	0.27	0.35*

* *p* < 0.05, ** *p* < 0.01, *** *p* < 0.001.

**TABLE 3:** Pearson correlation matrix between imagery subtest and perceptual and memory tasks—controls.

**Controls**	**Forward digit-span**	**Backward digit-span**	**Visual-spatial memory test**	**Memory of objects**	**Memory of position of objects**	**Mirror reproduction of spatial position**
Visualizing letters	0.26	–0.09	0.32*	0.16	0.10	0.24
Brooks “F” test	0.45***	0.30*	0.60***	0.48***	0.60***	0.10
Clock	–0.07	–0.04	0.08	–0.06	–0.01	0.01
Cube	–0.25	–0.07	0.29*	0.08	0.17	0.06
Subtraction of parts	0.31*	0.55***	–0.09	0.32*	0.04	0.32*
Mental exploration of a map	0.25	0.20	0.34*	0.42***	0.35**	0.31*
Imagined paths	0.49***	0.49***	0.46***	0.63***	0.57***	0.32*
Mental representation of shapes of objects	0.56***	0.61***	0.30*	0.61***	0.62***	0.57***

* *p* < 0.05, ** *p* < 0.01, *** *p* < 0.001.

Results showed that the mental imagery performances are differently correlated with perceptual and memory ones. As for group differences, an interesting exception to the general trend is the “clock” task, which is positively (although not significantly) correlated with memory and perception tasks in athletes, while in the control group, these correlations are null or inverse.

## Discussion

The present study aimed to assess the ability of children to engage in visual and spatial mental imagery (image maintenance, image inspection, image generation, and image manipulation), and to compare these processes between athletes and non-athletes.

Results showed high significant differences in all mental imagery abilities (except the “cube” test) and, in memory and visual perception with athletes. According to Moreau et al. (2011), imagery ability is known to be closely related to visual perception and to be determinant in learning, memory, and motor processes.

The only task showing a different trend is “clock”: the performance being better in the control group. This suggests that image maintenance abilities depend on stimulus complexity, according to previous research (e.g., Kosslyn, 1994; Frank et al., 2016). Children are better able to recall an image with specific features than an image with a more abstract pattern (Simonsmeier and Buecker, 2017). We, therefore, determine that the image generation task reasonably plays a significant role in reversal ability: the ability to form an image of another interpretation and impose this imagined assembly onto the figure.

Correlational analyses showed that the mental imagery performances are differently correlated with perceptual and memory ones. In the group of young athletes, the significant correlations are few and with lower size, indicating that in this group, the imagery functions are more independent from perception and memory than in non-athletes, and this independence could justify the overall better performance. We know that having solid mental imagery is an advantage to resolving visual and spatial tasks; nevertheless, our results propose that persons might use diverse cognitive approaches to resolve the similar visual memory task.

An interesting exception to this general trend is the “clock” task, which is positively correlated with memory and perception tasks in athletes (not significantly, although the size of the correlation with the forward digit span is relevant, i.e., 0.25), while in the control group, these correlations are null or inverse. In this task, the performance of the group of athletes is worse than that of the non-athletes; it could be influenced by the integration between memory and perceptual abilities, differently from controls. In fact, this task—being the most difficult—requires more than others to join visualizing and remembering the position in the space (of the hands of the visualized clock) and, therefore, an integration between imagery and other cognitive function, less present in the group of athletes as demonstrated in the other tasks. Probably, the young athletes are less able in the clock task for their less familiarity with analogical clocks, since they have more practice with digital clocks (e.g., for monitoring functioning during training and performances). For the same reason, in this task, the influence of the perceptual and memory abilities is more relevant in the athletes.

Despite the strengths of the current study, which include novel findings for a young population, there are several limitations that should be noted. For one, the ability of children to engage in visual and spatial mental imagery could be resulted with a longitudinal design. The relationship between the variables could be identified through since to start sport. Future research should investigate the mechanism more specifically, for example, investigating the association between cognitive-specific imagery ability and learning development of athletes.

## Conclusion

In conclusion, it can be determined that imagery seems to be an instinctive psychological ability, which grows in early years of life. This finding is in line with previous studies, which showed that children often use imagery to acquire skills in a natural way (Wolmer et al., 1999; Munroe-Chandler et al., 2007a). Consequently, to improve learning, performance, and other results related with imagery (such as self-efficacy, anxiety regulation, and cognition), athletes and coaches should incorporate the regular practice of mental imagery in their training programs.

## Data Availability Statement

All datasets generated for this study are included in the article/Supplementary Material.

## Ethics Statement

The studies involving human participants were reviewed and approved by the University Enna Kore Internal Review Board for psychological research (September 26, 2019). Written informed consent to participate in this study was provided by the participants’ legal guardian/next of kin.

## Author Contributions

DD, MG, SD, and MC contributed to the conception and design of the study. CG and SC were responsible for testing. MC, MG, SD, DD, and NM were responsible for data collection and statistical analysis. DD, MG, and MC were responsible for drafting and finalization of the manuscript. All authors contributed to manuscript revision and approved the submitted version.

## Conflict of Interest

The authors declare that the research was conducted in the absence of any commercial or financial relationships that could be construed as a potential conflict of interest.

## Supplementary Material

DATA SHEET S1 Datasets for this study.
